# Removal of melatonin receptor type 1 signalling induces dyslipidaemia and hormonal changes in mice subjected to environmental circadian disruption

**DOI:** 10.1002/edm2.171

**Published:** 2020-09-10

**Authors:** Cynthia Tchio, Kenkichi Baba, Giuseppe Piccione, Gianluca Tosini

**Affiliations:** ^1^ Circadian Rhythms and Sleep Disorders Program Neuroscience Institute Atlanta GA USA; ^2^ Department of Pharmacology and Toxicology Morehouse School of Medicine Neuroscience Institute Atlanta GA USA; ^3^ Dipartimento di Medicine Veterinaria Universita di Messina Messina Italy

**Keywords:** environmental circadian disruption and lipids, female, male, melatonin‐proficient mice, MT_1_ signalling

## Abstract

**Background:**

Melatonin is a hormone secreted by the pineal gland in a circadian rhythmic manner with peak synthesis at night. Melatonin signalling was suggested to play a critical role in metabolism during the circadian disruption.

**Methods:**

Melatonin‐proficient (C3H‐f^+/+^ or WT) and melatonin receptor type 1 knockout (MT_1_ KO) male and female mice were phase‐advanced (6 hours) once a week for 6 weeks. Every week, we measured weight, food intake and basal glucose levels. At the end of the experiment, we sacrificed the animals and measured the blood's plasma for lipids profile (total lipids, phospholipids, triglycerides and total cholesterol), metabolic hormones profiles (ghrelin, leptin, insulin, glucagon, glucagon‐like‐peptide and resistin) and the body composition.

**Results:**

Environmental circadian disruption (ECD) did not produce any significant effects in C3H‐f^+/+^, while it increased lipids profile in MT_1_ KO with the significant increase observed in total lipids and triglycerides. For metabolic hormones profile, ECD decreased plasma ghrelin and increased plasma insulin in MT_1_ KO females. Under control condition, MT_1_ KO females have significantly different body weight, fat mass, total lipids and total cholesterol than the control C3H‐f^+/+^ females.

**Conclusion:**

Our data show that melatonin‐proficient mice are not affected by ECD. When the MT_1_ receptors are removed, ECD induced dyslipidaemia in males and females with females experiencing the most adverse effect. Overall, our data demonstrate that MT_1_ signalling is an essential modulator of lipid and metabolic homeostasis during ECD.

## Graphical abstract

Melatonin‐proficient mice are protected against environmental circadian disruption (ECD), while removal of melatonin receptor type 1 (MT_1_) signalling induces dyslipidaemia in males and females and hyperinsulinaemia in only MT_1_ knockout (MT_1_ KO) females. Our experiments suggest that MT_1_ KO female mice are more affected by ECD than their male counterpart, thus suggesting that ECD may have more harmful effects on females.

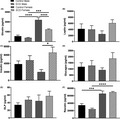

## 1 INTRODUCTION

Melatonin is a chronobiotic hormone secreted by the pineal gland in a rhythmic manner that reaches peak circulating levels during the night.^1^ Once melatonin is synthesized, it exerts its function via two G‐protein‐coupled receptors named melatonin receptor type 1 (MT_1_) and melatonin receptor type 2 (MT_2_).^2^, ^3^ MT_1_ and MT_2_ are both expressed in the mammalian brain master clock—suprachiasmatic nuclei (SCN) of the hypothalamus,^2^, ^4^, ^5^ and both receptors are involved in the entrainment of circadian rhythms by SCN.^6^ During the circadian disruption, it is still unclear which one of the two receptors is actually responsible for the entrainment of the circadian rhythms in locomotor activity.^7^ Experimental evidence suggests that MT_1_ is required for the melatonin‐induced phase‐shift of the circadian rhythm in locomotor activity ^8^. Whereas, MT_2_ is implicated in the re‐entrainment of the circadian rhythm of locomotor activity.^9^

Recent studies have also implicated melatonin signalling in the regulation of metabolism and glucose homeostasis.^10^, ^11^, ^12^ Our laboratory has shown that mice lacking MT_1_ exhibit a marked systemic insulin resistance while MT_2_ knockout (KO) did not^13^; additionally, we also found that MT_1_ KO mice also tend to accumulate more fat mass than the control mice.^14^ We also reported that MT_1_ KO mice exhibit a selective leptin resistance in the arcuate nucleus.^15^ Moreover, MT_1_ KO mice subjected to a high‐fat diet also showed a significant increase in cumulative weight gain and basal glucose levels (>200 mg/dL) after 10 weeks.^11^ Interestingly in our previous study, we did not observe any phenotype in mice lacking MT_2_.^11^, ^13^

Previous studies have shown that environmental circadian disruption (ECD) has severe consequences on the mouse's overall health. In a seminal study by Davidson et al, it was reported that chronic jet‐lag (6‐hour advance for 8 weeks) increases mortality in aged mice.^16^ Additional studies indicated that ECD dysregulated the innate immune system independently from sleep loss or stress ^17^ and led to an increase in body weight, adipocytes size and triglycerides concentration.^18^, ^19^, ^20^, ^21^ However, it is worth noting that all these experiments have been performed only in mice males on a C57/Bl6 genetic background, a melatonin‐deficient strain.^22^, ^23^

The aim of the present study was first to investigate the effects of ECD in melatonin‐proficient mice strain (C3H‐f^+/+^) and then to determine the effects of ECD in C3H‐f^+/+^ mice lacking MT_1_ receptors.

## 2 MATERIALS AND METHODS

### 2.1 Animals and housing conditions

Melatonin‐proficient mice (C3H‐f^+/+^ wild type), melatonin‐proficient mice lacking MT_1_ (C3H‐f^+/+^‐MT_1_ KO), were used in our study.^13^ All mice were 3 months old at the beginning of the experiment. Mice were single caged, and water and chow (5L0D Picolab^®^ laboratory rodent diet; LabDiet) were available ad libitum. All experimental procedures were performed under the NIH Guide on Care and Use of Laboratory Animals and were approved by the Morehouse School of Medicine Animal Care and Use Committee ^24^, ^25^, ^26^.

### 2.2 Environmental circadian disruption

**FIGURE 1:**
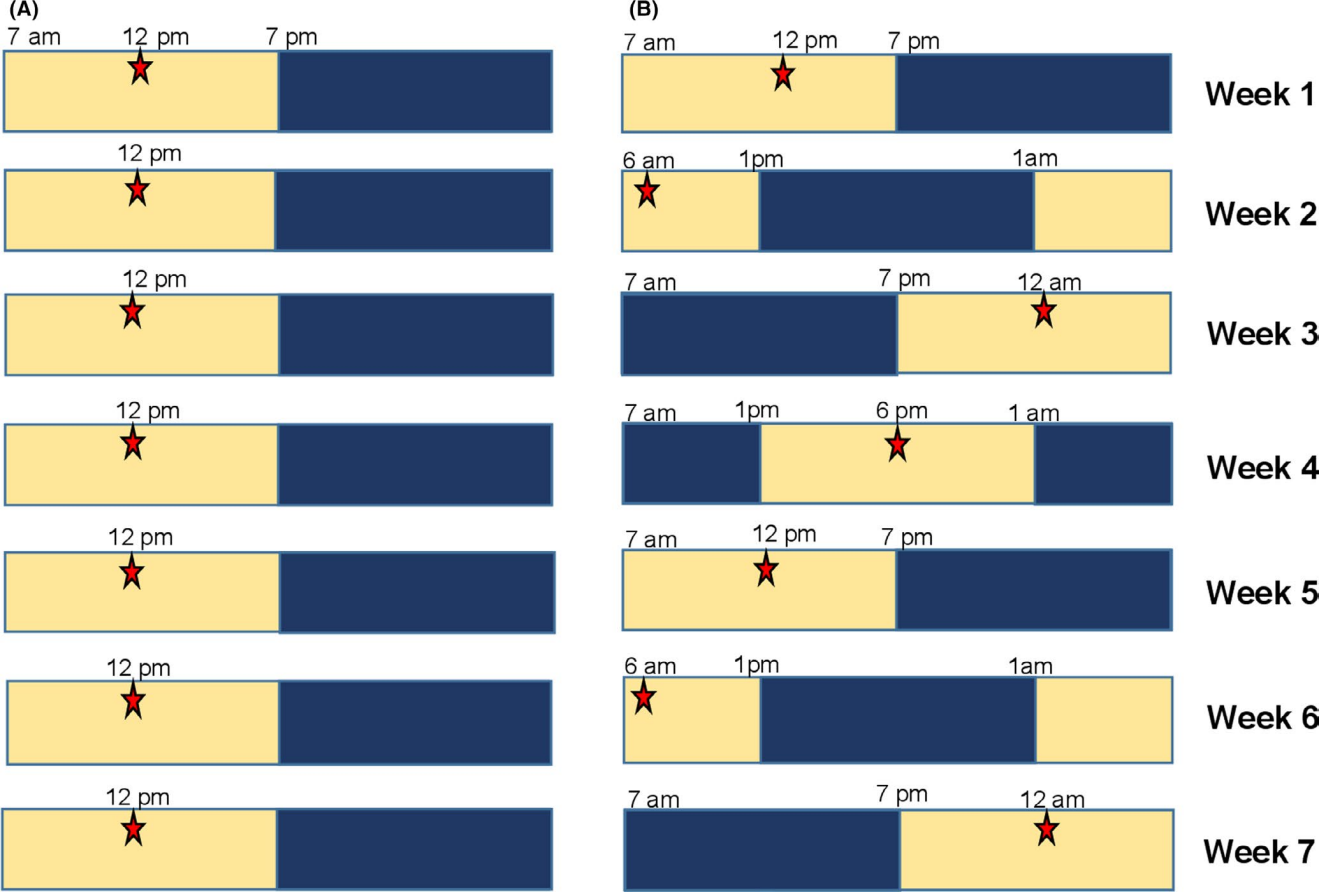
Environmental circadian disruption (ECD) light schedule. A, Control animals were under 12:12 light and dark (LD) cycle where the lights turn on at 7 am and turn off at 7 pm; for a period of 7 wk. B, ECD animals were under 12:12 light and dark cycle where the lights turn on at 7 am and turn off at 7 pm for week1; the LD was 6‐h phase advance for the following week (week 2), and the mice were given 7 d to adjust to new LD before the next phase advance; this was repeated for a total period of 7 wk. On the 6th days of the week, the mice fasted for 5 h when the light was turned on and the stars represent the time at which body parameters were measured

Control mice were maintained under the 12:12 light‐dark cycle while the ECD groups were maintained under the 6‐hour phase advance schedule described in Davidson et al^16^ (Figure 1). The ECD group were maintained under ECD for 6 weeks, All mice were sacrificed and the plasma was collected at 12:00 pm on the 6th day after the last shift.

### 2.3 Body composition

At the end of the experiment, the mice were anaesthetized with isoflurane; then, we performed a cardiac puncture for the blood collection. After the blood collection, the mice were sacrificed, and the body composition analysis was performed at the University of Cincinnati National Mouse Metabolic Phenotyping Center,^27^ using quantitative magnetic resonance.

### 2.4 Lipid analysis

Blood was collected and treated with 100 μL of 0.5 mol/L of ethylenediaminetetraacetic acid. The treated blood was centrifuged at 2000 *g* for 15 minutes at 4°C, and the plasma (supernatant) was frozen and stored at −80°C. Lipids profile protocol is described in detail here.^28^

### 2.5 Metabolic hormones plasma analysis

The plasmas' level of metabolic hormones (ghrelin, leptin, insulin, glucagon, glucagon‐like‐peptide and resistin) were measured using a commercially available suspension of magnetic bead array immunoassay kit following manufacturer's specifications (BIO‐RAD Bio‐Plex Pro Mouse Diabetes 8‐Plex Assay #171f7001m).

### 2.6 Data analysis

Results are presented as mean ± SEM. The significance level was set at *P* = .05 with a power >0.8. Statistical analyses were performed using two‐way analysis of variance followed post hoc Tukey test in GraphPad Prism version 8.0.

## 3 RESULTS

### 3.1 Effects of melatonin signalling removal under normal lighting conditions

Our data indicate that under normal lighting conditions, several differences are present among the different genotypes and sex. C3H‐f^+/+^ females have significantly higher body weight, fat mass, total lipids and total cholesterol than MT_1_ KO female (Two‐way ANOVA Followed by Tukey test, *P* < .05) whereas C3H‐f^+/+^ males have significantly lower levels of phospholipids than MT_1_ KO males (two‐way ANOVA Followed by Tukey test, *P* < .05). Basal glucose levels were also slightly lower in C3H‐f^+/+^ males than in MT_1_ KO males (two‐way ANOVA followed by Tukey test, *P* < .05). Finally, ghrelin levels were significantly lower in C3H‐f^+/+^ females than in MT_1_ KO females (two‐way ANOVA followed by Tukey test, *P* < .05).

### 3.2 Melatonin‐proficient mice are protected from ECD physiological change

**FIGURE 2:**
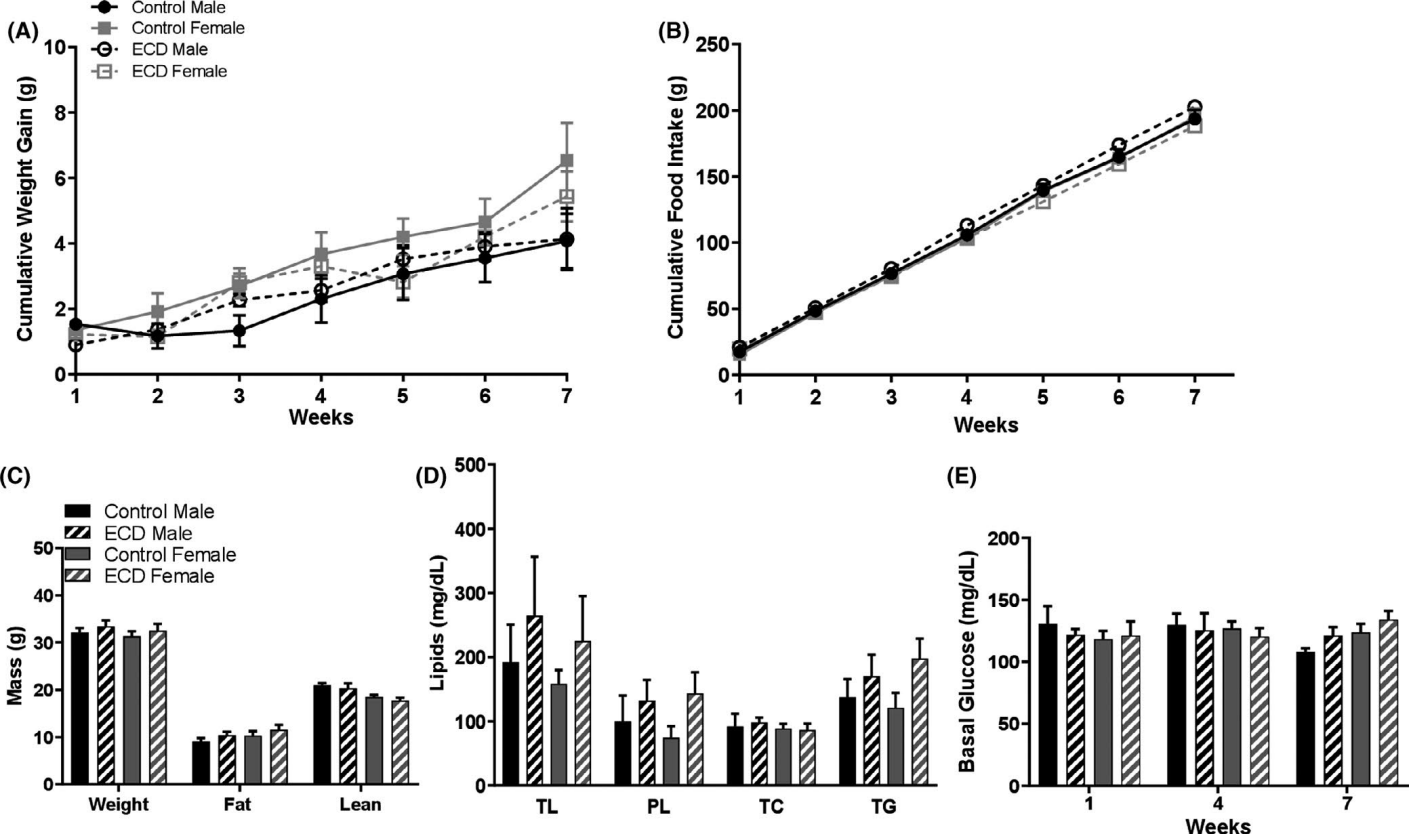
Melatonin‐proficient wild‐type mice are protected against environmental circadian disruption (ECD)‐associated dysfunctions. A, Cumulative weight gain; two‐way ANOVA repeated measured followed by Tukey post hoc test. B, Cumulative food intake; two‐way ANOVA repeated measured followed by Tukey post hoc test. C, Body composition; two‐way ANOVA and followed by Tukey post hoc test D, Lipids composition: total lipids (TL), phospholipids (PL), total cholesterol (TC), triglycerides (TG); two‐way ANOVA and followed by Tukey post hoc test. E, Fasting blood glucose; two‐way ANOVA repeated measured followed by Tukey post hoc test. Results are expressed in mean ± SEM (n = 6‐8)

**FIGURE 3:**
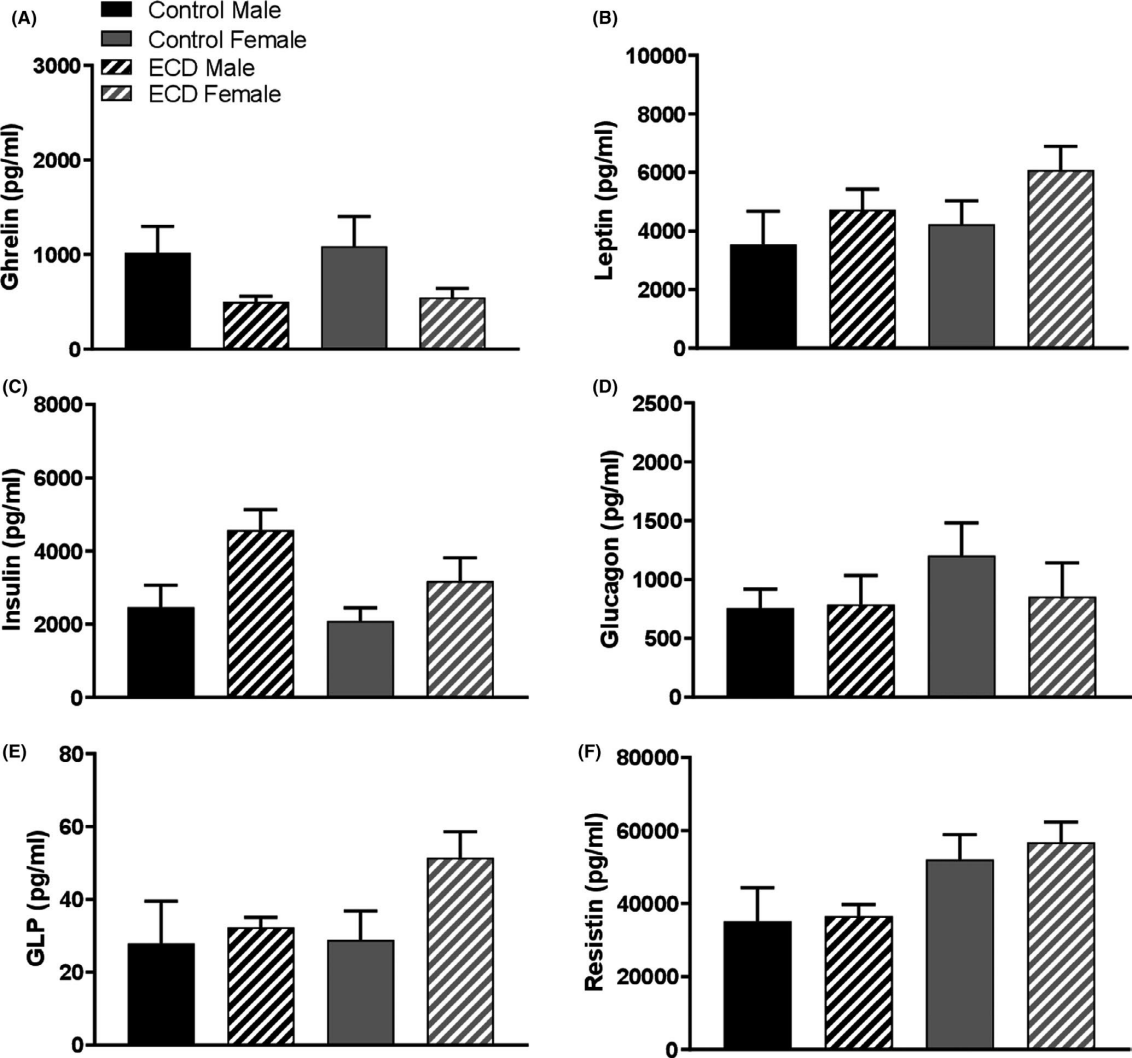
Melatonin‐proficient wild‐type mice are protected against environmental circadian disruption (ECD) dysregulation of metabolic hormones. A, Ghrelin (B) leptin (C) insulin (D) glucagon (E) glucagon‐like peptide (GLP) (F) resistin. Results are expressed in mean ± SEM (n = 6‐8; two‐way ANOVA followed by Tukey post hoc test)

Figure 2 displays the results obtained in C3H‐f^+/+^ mice under the control condition and ECD. Although females showed a tendency to an increase in cumulative weight, no significant differences were observed in all the parameters investigated between males and females and between control and ECD mice (Figure 2A‐E; *P* > .05 in all cases). No difference among the different groups was also detected in the plasma levels of the six metabolic hormones (Figure 3A‐F, *P* > .05 in all cases).

### 3.3 Removal of MT_1_ signalling induces dyslipidaemia and hormonal changes in mice subjected to ECD

**FIGURE 4:**
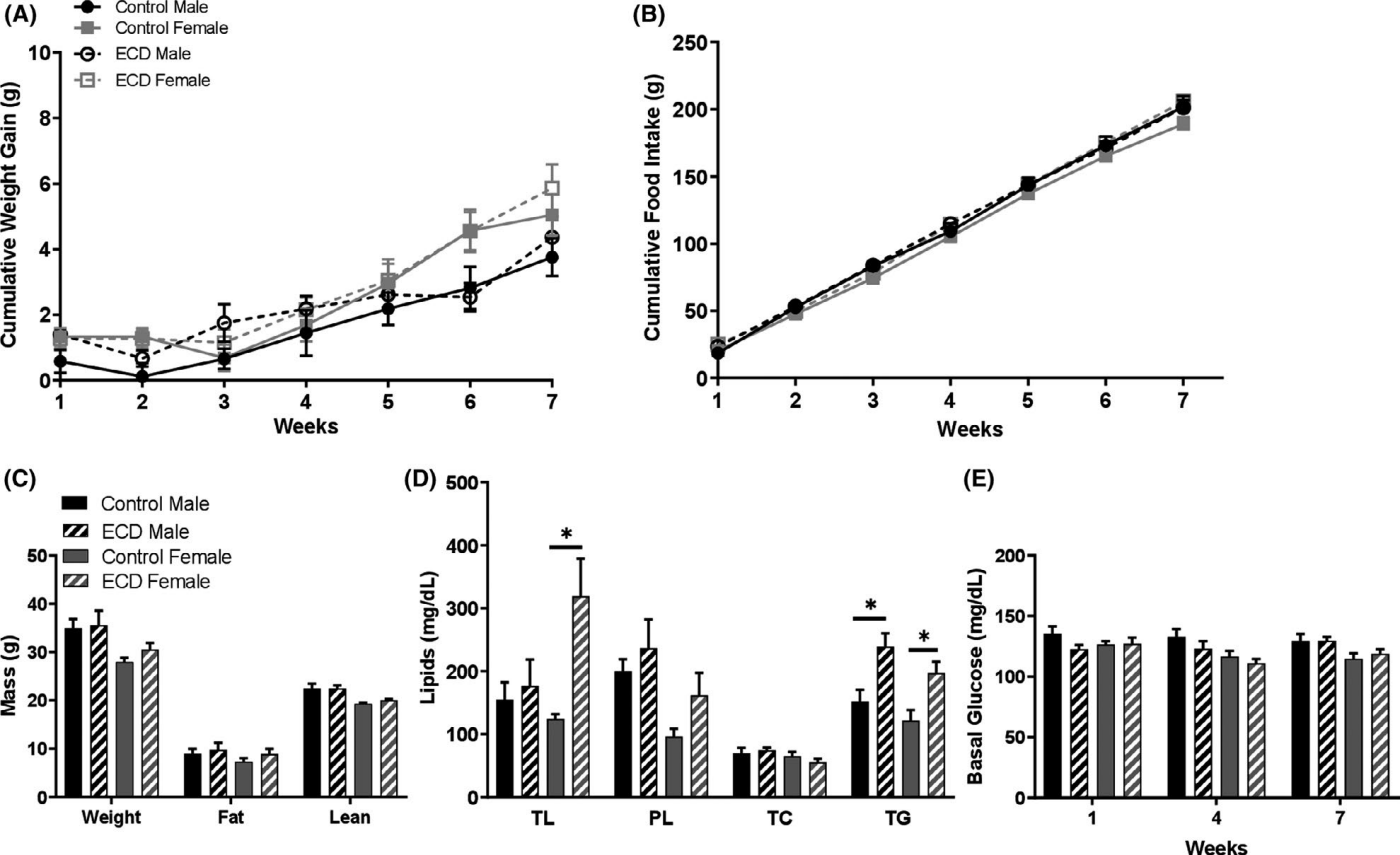
Environmental circadian disruption (ECD) induces dyslipidaemia in MT_1_^−/−^ male and female. A, Cumulative weight gain; two‐way ANOVA repeated measured followed by Tukey post hoc test. B, Cumulative food intake; two‐way ANOVA repeated measured followed by Tukey post hoc test. C, Body composition; two‐way ANOVA and followed by Tukey post hoc test. D, Lipids composition: total lipids (TL), phospholipids (PL), total cholesterol (TC), triglycerides (TG); two‐way ANOVA and followed by Tukey post hoc test,*P < .05. E, Fasting blood glucose; two‐way ANOVA repeated measured followed by Tukey post hoc test. Results are expressed in mean ± SEM (n = 6‐8)

**FIGURE 5:**
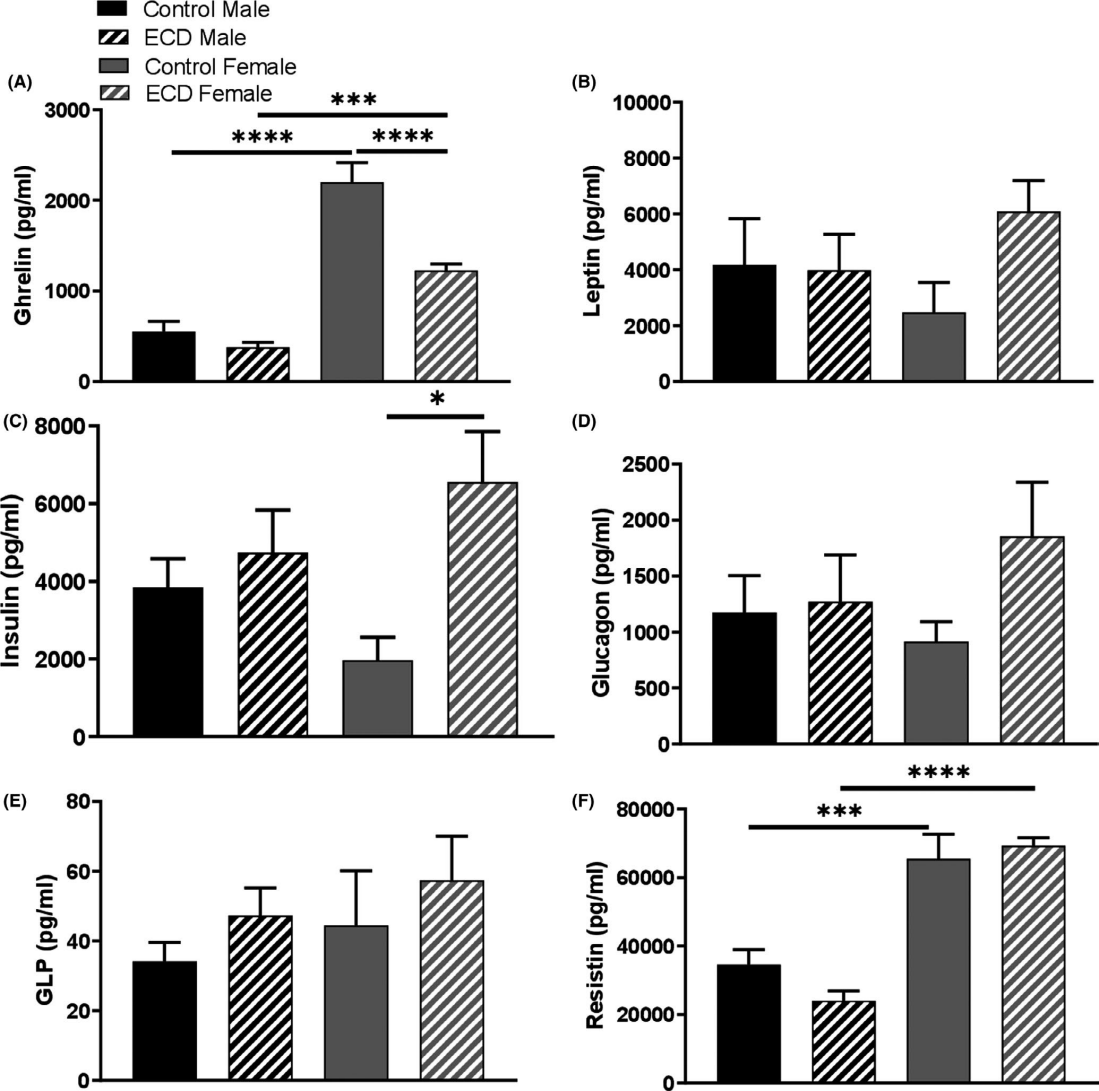
Environmental circadian disruption (ECD) decreases plasma ghrelin and increases plasma insulin in MT_1_^−/−^ female. A, Ghrelin (B) leptin (C) insulin (D) glucagon (E) glucagon‐like peptide (GLP) (F) resistin. Results are expressed in mean ± SEM (n = 6‐8; two‐way ANOVA followed by Tukey post hoc test, *P < .05, ***P < .001**,** ****P < .0001)

Figure 4 shows that the results obtained in MT_1_ KO mice under control and ECD conditions. No significant difference was observed in most of the parameter investigated (Figure 4A‐C,E, *P* > .05), but a significant increase in total lipids (almost 3 times with respect to control conditions) was observed in the female under ECD (Figure 4D; *P* < .05), and a significant increase in triglycerides levels was observed in both male and female mice (Figure 4E; *P* < .05). Under control conditions, MT_1_ KO females showed a significant increase in ghrelin and resistin levels with respect to the values measured in MT_1_ KO males (Figure 5A,F; *P* < .05). ECD significantly reduced the levels of ghrelin (Figure 5A; *P* < .05) and increased the level of Insulin (Figure 5C; *P* < .05) in MT_1_ KO females.

## 4 DISCUSSION

Disruption of melatonin synthesis and/or signalling is believed to be a co‐factor in the development of metabolic disorders in the general population^29^, ^30^ and in shift workers.^31^, ^32^ Although many studies have investigated the effects of ECD in mice, most of these studies have been performed in mice strains that do not synthesize melatonin, and no study has considered the effects of sex. In this study, we have investigated the effects of ECD in males and females' melatonin‐proficient mice model. From our research, we found that ECD did not induce any significant change in these mice, thus suggesting that melatonin signalling is exerting a protective effect against ECD. The removal of MT_1_ abolished the melatonin protection, and MT_1_ KO females seem to be more susceptible to ECD than the males.

As we have previously mentioned, our laboratory has reported that mice lacking MT_1_ show insulin and leptin resistance,^14^, ^15^ and we thus investigated whether ECD will further negatively affect the MT_1_ KO metabolic phenotypes.^11^, ^14^, ^15^ Our data show that MT_1_ KO male and female mice showed an increase in plasma lipids with a significant increase in total lipids and triglycerides (Figure 4D). Such a result is as we expected because it has been reported that the administration of exogenous melatonin can reduce serum and liver triglycerides in diabetic male rats and mice ^33^, ^34^ and in obese C57BL6 male mice.^35^ Therefore, our data indicate that the activation of MT_1_ signalling exerts a protective effect on lipids concentration in mice subjected to ECD.

We also observed that ECD decreased the ghrelin level in MT_1_ KO with a significant decrease in MT_1_ KO females. Ghrelin is an orexigenic hormone secreted by the gut that binds to the growth hormone secretagogue receptor (GHS‐R).^36^ The serum ghrelin level peaks during the resting phase and is mainly regulated by food intake.^37^, ^38^ Some studies have also reported that melatonin also decreases ghrelin levels in dogs and rats,^39^, ^40^ while in humans, plasma melatonin concentration correlated negatively with ghrelin, thus suggesting a possible role for melatonin in the regulation of ghrelin concentration.^41^ Ghrelin is also a key regulator in glucose homeostasis, and it regulates insulin secretion via heterodimerization of G‐protein‐coupled receptor GSH‐R and somatostatin‐5^42^; this could explain why MT_1_ KO females have an increase in plasma insulin after ECD. Oestrogen is reported to protect mammalian females against obesity, and circadian disruption is associated with metabolic dysfunction,^43^, ^44^, ^45^ which might suggest that ECD has a more significant disruptive effect on oestrogen signalling in melatonin‐proficient MT_1_ KO female.

Our data also indicate that MT_1_ KO females have a higher plasma concentration of resistin when compared to MT_1_ KO males regardless of the treatment group. This is of interest because resistin is a hormone secreted by the white adipose whose name came from inhibiting insulin sensitivity, thus resisting insulin.^46^, ^47^ Loss of resistin was reported to improve glucose homeostasis,^48^ and resistin was also reported to play a role in obesity via AMPKα and acetyl‐CoA carboxylase ^49^, ^50^, ^51^. In our C3H‐f^+/+^ mice, there was no significant difference in plasma resistin between males and females. Thus, it appears that the removal of MT_1_ signalling affects the resistin level in a sex‐specific manner. Melatonin supplementation was reported to improve obesity‐induced resistin elevation.^35^, ^52^, ^53^ Stubbins et al found that oestrogen protects female mice against obesity and impaired glucose tolerance, furthermore, they also report that oestrogen lowers the circulating resistin level.^43^ Subsequent studies are needed to explore the mechanism of the effect of MT_1_ melatonin signalling on metabolic hormones in the female melatonin‐proficient mice. On the other note, it was observed that female shift workers experienced more stress and greater intolerance to the shift schedule than male workers.^54^, ^55^ Melatonin was reported to improve the subjects’ sleep quality in a simulated shift night study.^56^ Thus, our findings are highlighting the need for studies that will further explore the mechanistic role of MT_1_ signalling in female circadian and metabolic homeostasis.

In conclusion, our results indicate that melatonin‐proficient mice are protected against ECD, while the removal of MT_1_ signalling induces dyslipidaemia in males and females and hyperinsulinaemia in only MT_1_ KO females. Similarly, it was observed that female shift workers experienced more stress and greater intolerance to the shift schedule than male workers. Our experiments also suggest that MT_1_ KO female mice are more affected by ECD than their male counterpart, thus suggesting that ECD may have more harmful effects on females.

## CONFLICT OF INTEREST

The author(s) declare that they have no conflict of interests.

## AUTHOR CONTRIBUTIONS

CT, KB, GP and GT designed the experiments, CT and GP performed the experiments, CT, KB, GP and GT analysed the data, CT wrote the first draft of manuscript that was edited and by all co‐authors.

## Data Availability

The original data are available upon request from the corresponding author.
